# The Relationship Between Oxidative Stress and Infertility Due to Antihypertensive Drugs in Rattus Norvegicus

**DOI:** 10.3390/ani14243674

**Published:** 2024-12-20

**Authors:** Berna Asir, Yakup Kumtepe

**Affiliations:** 1 Department of Obstetrics and Gynecology, Erzurum City Hospital, Erzurum 25030, Turkey; 2 Department of Obstetrics and Gynecology, Ankara University, Ankara 06100, Turkey; ykumtepe@hotmail.com

**Keywords:** antihypertensive drugs, antioxidant effect, infertility, oxidative stress

## Abstract

This study aimed to investigate the effect of antihypertensive drugs on reproductive function in Rattus norvegicus and demonstrate the potential role of oxidative stress in reproductive dysfunction. Rattus norvegicus were selected as the experimental animals and divided into the following groups: healthy (control group), clonidine (CL), rilmenidine (RLD), methyldopa (MTL), amlodipine (ALD), and ramipril (RML). Each individual in each group was marked from one to six. Doses of clonidine (0.075 mg/kg), rilmenidine (0.5 mg/kg), methyldopa (100 mg/kg), amlodipine (2 mg/kg), and ramipril (2 mg/kg) were administered orally via gavage to each Rattus norvegicus. Using blood obtained from Rattus norvegicus, the absorbance of the pink-colored complex formed by thiobarbituric acid (TBA) and malondialdehyde (MDA) was measured spectrophotometrically at the 532 nm wavelength. Blood samples were collected from the tail veins to analyze serum malondialdehyde (MDA) and total glutathione levels in the serum of all Rattus norvegicus. After sampling, two mature male Rattus norvegicus were introduced to every group of six female Rattus norvegicus and accommodated in a controlled laboratory environment for two months. Any female Rattus norvegicus that became pregnant during this time was transferred to a solitary cage within a controlled setting. Rattus norvegicus that did not become pregnant and did not give birth during this period were considered infertile. The results were compared among the groups. Total glutathione (tGSH) levels were determined using a spectrophotometer. According to our study, the increase in MDA levels observed was not statistically significant in the CL and RLD groups compared to that in the control group. MDA levels were significantly increased in the methyldopa, amlodipine, and RML groups. While total glutathione levels in the CL group were similar to those in the control group, the RLD, MTL, ALD, and RML groups showed a statistically significant decrease. While the animals in the CL and RLD groups were not infertile, infertility was apparent in the groups treated with MTL, ALD, and RML. Thus, it was determined that the antihypertensive drugs MTL, ALD, and RML had different effects on fertility, and that the use of such drugs could cause infertility by increasing oxidative stress and decreasing antioxidant levels.

## Simple Summary

We aimed to investigate the effect of antihypertensive drugs on reproductive function in Rattus and demonstrate the role of oxidative stress in reproductive dysfunction. Rattus subjects were divided into six groups. Doses of clonidine, rilmenidine, methyldopa, amlodipine, and ramipril were administered orally via gavage to each Rattus subject. Blood samples were collected from the tail veins. After sampling, two mature male Rattus subjects were introduced to every group. Any female Rattus subjects that became pregnant during this time were transferred to a solitary cage within a controlled setting. Rattus subjects that did not become pregnant and did not give birth during this period were considered infertile. In our study, the increase in MDA levels observed was not statistically significant in the CL and RLD groups compared to those in the control group. MDA levels were significantly increased in the methyldopa, amlodipine, and RML groups. While total glutathione levels in the CL group were similar to those in the control group, the RLD, MTL, ALD, and RML groups showed a statistically significant decrease. Thus, it was determined that the antihypertensive drugs MTL, ALD, and RML had different effects on fertility and that the use of such drugs could cause infertility by increasing oxidative stress and decreasing antioxidant levels.

## 1. Introduction

Infertility is defined as the failure to establish a clinical pregnancy despite having regular sexual intercourse for at least one year without using contraceptive methods [1]. The causes of infertility include decreased ovarian reserve; dysregulated ovulation; tubal, uterine, pelvic, and male factors; and unexplained reasons [2].

In the literature, it is asserted that oxidative stress has a role in the pathogenesis of many diseases that can cause infertility (ovarian ischemia–reperfusion injury, polycystic ovarian syndrome, infection, endometriosis, etc.) [3,4,5,6]. Furthermore, the long-term use of chemotherapeutic agents during childhood and when at a reproductive age may cause oxidative stress, which is associated with ovarian failure and infertility [7,8]. Likewise, it is reported that exposure to environmental factors, such as organochlorine compounds, perfluorochemicals, and cigarette smoke, can cause infertility by inducing oxidative stress [6]. Similarly, it has been argued that women with chronic hypertension are at an increased risk of infertility [9,10].

Several antihypertensive drugs are used to prevent hypertension and avoid related complications. Diuretics, sympatholytics or adrenergic nervous system antagonists, medications that modulate vascular smooth muscle, and drugs affecting the renin−angiotensin system (RAS) are all used in the treatment of hypertension. Beta-blockers, diuretics that are commonly prescribed to treat hypertension, can reduce blood flow to the uterus and affect implantation, thus impeding conception. Several antihypertensive drugs have been widely studied for their systemic effects; however, their reproductive implications remain underexplored. Clonidine, a central alpha-2 adrenergic agonist, is primarily used for its central blood pressure-lowering effects, whereas rilmenidine acts on imidazoline receptors to modulate sympathetic activity [11]. Methyldopa, a prodrug converted into an alpha-2 adrenergic agonist, is commonly used during pregnancy, although it has notable potential oxidative effects [12]. Amlodipine, a calcium channel blocker, is frequently prescribed due to its efficacy in reducing vascular resistance; however, it has also been associated with the moderation of oxidative stress in ovarian tissues. The angiotensin-converting enzyme (ACE) inhibitor ramipril is effective in managing hypertension; however, there are concerns regarding the risk of severe oxidative stress-induced ovarian damage [13]. Understanding the specific oxidative mechanisms of antihypertensive drugs is crucial for evaluating their impact on fertility and reproductive health.

Several antihypertensive medications are known to have serious side effects, including sedation, tremor (methyldopa), fetal development delay (atenolol and metoprolol), intrauterine developmental delay (labetalol), neonatal thrombocytopenia (hydralazine), edema, flushing (calcium channel blockers), and fetal death [14,15,16,17]. In one animal study, antihypertensive drugs, including clonidine and rilmenidine, did not induce a significant change in the oxidant and antioxidant parameters of the ovarian tissue. However, methyldopa did cause mild oxidative damage, and amlodipine and ramipril caused moderate and severe oxidative stress, respectively, resulting in ovarian damage [13]. According to the literature, it is apparent that antihypertensive drugs that cause oxidative stress in ovarian tissues may promote reproductive dysfunction [11,12,18]. However, no studies in the literature have specifically analyzed the effect of clonidine, rilmenidine, methyldopa, amlodipine, and ramipril on animal reproductive function. Therefore, in our study, we aimed to investigate the effects of clonidine, rilmenidine, methyldopa, amlodipine, and ramipril on reproductive function in rats and to determine the role of oxidative stress in potential reproduction dysfunction.

## 2. Materials and Methods

**Selection of experimental animals:** For our experiments, 36 adult albino Wistar species female Rattus norvegicus with a weight of 245–267 g were used. Rattus norvegicus were obtained from the Atatürk University Medical Experimental Application and Research Center laboratories and kept in groups at room temperature (22 °C).

**Experimental procedure:** Experimental animals were divided into healthy (control), clonidine (CL), rilmenidine (RLD), methyldopa (MTL), amlodipine (AML), and ramipril (RML) groups. The Rattus norvegicus in each group were marked with a number from one to six. Doses of clonidine (0.075 mg/kg), rilmenidine (0.5 mg/kg), methyldopa (100 mg/kg), amlodipine (2 mg/kg), and ramipril (2 mg/kg) were administered orally via gavage to the Rattus norvegicus, referred to as CL (n = 6), RL (n = 6), MTL (n = 6), ALD (n = 6) and RML (n = 6), respectively. Drug dosage adjustment was determined according to the information available in the literature and was dependent on the weight of the rats [12]. The control group (n = 6) was administered an equivalent volume of distilled water. This procedure was repeated once each day for thirty days. Following this, blood samples were collected from the tail veins to analyze serum malondialdehyde (MDA) and total glutathione levels in the serum of all Rattus norvegicus. After sampling, two mature male Rattus norvegicus were introduced into each group of six female Rattus norvegicus and kept in a controlled laboratory environment for two months. Pregnant Rattus norvegicus were transferred to a solitary cage within a controlled setting during this time. Rattus norvegicus that did not become pregnant or did not give birth during this period were considered infertile. The results were evaluated and compared among the groups.

### Biochemical Analyses

**Preparation of the samples:** Blood samples were drawn from all Rattus norvegicus and transferred into serum tubes containing separation gels. All the blood samples were incubated for 15 min at room temperature. The serum was separated by centrifugation at 1500× *g* for 10 min. All serum samples were stored at −80 °C until biochemical analysis.

**Serum MDA levels:** Serum MDA levels were evaluated at the Atatürk University Medical Biochemistry Department Research Laboratory using the MDA level evaluation method described by Okhawa et al. This technique involves measuring the absorbance of the pink-colored complex formed by thiobarbituric acid (TBA) and MDA at a high temperature (95 °C) using a spectrophotometer at a wavelength of 532 nm [18].

**Experimental procedure:** A 0.1 mL aliquot of the serum sample was added to a solution containing 0.2 mL 80 g/L sodium dodecyl sulfate, 1.5 mL 200 g/L acetic acid, 1.5 mL 8 g/L 2-thiobarbiturate, and 0.3 mL distilled water. The solution was incubated for one hour at 95 °C. After cooling, 5 mL of n-butanol/pyridine (15:1) was added. The solution was mixed using a vortex mixer and centrifuged for 30 min at 4000 rpm. The absorbance of the supernatant was measured at a wavelength of 532 nm. A standard curve was obtained using 1,1,3,3- tetra methoxy propane.

**Serum tGSH levels:** Serum GSH levels were evaluated using a spectrophotometer according to the method described by Sedlak and Lindsay. The chromogen DTNB (5 5′-dithiobis [2-nitrobenzoic acid]) disulfide is easily reduced by sulfhydryl group compounds, and the yellow color produced during reduction is measured using spectrophotometry at a wavelength of 412 nm.

**Experimental Procedure:** Prior to performing the measurements, a cocktail solution (buffer solution) was prepared (5.85 mL 100 mM phosphate-buffered saline, 2.8 mL 1 mM DTNB, 3.75 mL 1 mM NADPH, and 80 uL 625 U/L glutathione reductase), then 0.1 mL metaphosphoric acid was added to 0.1 mL serum to induce deproteinization, and the solution was centrifuged for 2 min at 2000 rpm. Finally, 0.15 mL cocktail solution was added to 50 uL supernatant. A standard curve was constructed using oxidized glutathione (GSSG). The yellow color formed was measured against distilled water at a wavelength of 412 nm using a spectrophotometer.

**Statistical Analysis: Experiment results** are presented as “mean value ± standard deviation”. The conformity of the parameters to the normal distribution was evaluated using the Kolmogorov–Smirnov test. The degree of importance between intergroup differences was determined using a one-way ANOVA test and an independent samples *t*-test. All statistical analyses were conducted using “IBM SPSS Statistics Version 20” (SPSS, Chicago, IL, USA), and a *p*-value <0.05 was accepted as significant.

## 3. Results

### 3.1. Experiment Procedural Findings

The results generated during the execution of the procedures detailed in the previous section are as follows:

No infertile Rattus norvegicus were present in the control group, and an average of six offspring were born in this group. The mean pregnancy duration for Rattus norvegicus in the control group was 30 days. The mean pregnancy duration for Rattus norvegicus treated with clonidine (CL group) was 33 days, and a similar number of offspring to the control group were produced. The mean pregnancy duration in the RDL group was 32 days, and the mean number of offspring in this group was six. All Rattus norvegicus in the control, CL, and RLD groups were fertile. One Rattus norvegicus specimen from the MTL group was infertile. The remaining Rattus norvegicus in this group had a 34-day average pregnancy duration. The average number of offspring in the MTL group was six, excluding the infertile rat. The number of pregnant Rattus norvegicus rats in the ALD group was four, and the number of infertile rats in the group was two. The fertile Rattus norvegicus in this group had an average pregnancy duration of 38 days and produced five offspring. The rats in the RML group had the highest infertility rate; three Rattus norvegicus were infertile and three were fertile, with an average pregnancy duration of 45 days. The number of offspring produced on average was four (Table 1).

### 3.2. Results of Biochemical Analysis

**Figure 1:**
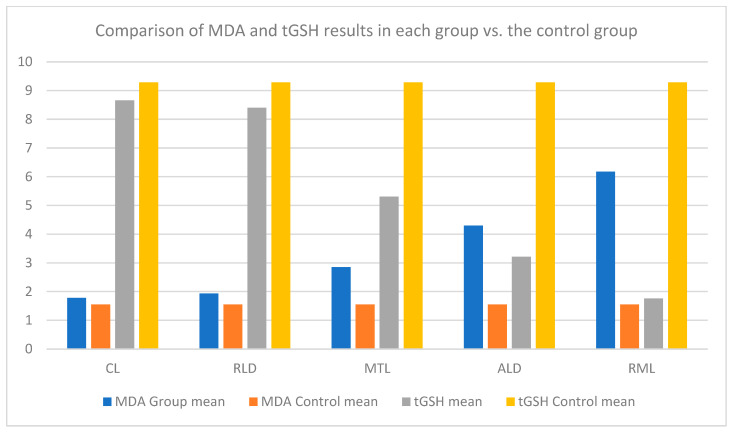
Comparison of MDA and tGSH results in each group vs. the control group.

**Serum MDA levels:** MDA and tGSH levels were measured in serum obtained from Rattus norvegicus. As shown in the results presented in Table 2, the RML group rats had the highest serum MDA levels. The lowest serum MDA level was recorded in the control group. According to the results presented in Figure 1, the control group had the highest tGSH levels among all groups, and the RML group had the lowest tGSH levels.

## 4. Discussion

Trends in the desire to have children have shifted toward people having children at the end of the reproductive period due to changes in socioeconomic circumstances, the increased accessibility of university education, career plans, etc. As a result, the prevalence of both infertility and hypertension has increased, and the concomitant need for antihypertensive medication has escalated. In this study, we assessed common antihypertensive medications, including clonidine, rilmenidine, methyldopa, amlodipine, and ramipril. Prior studies have investigated the effects of these medications on the oxidant and antioxidant parameters of uterine and ovarian tissues and found that these medications can cause varying degrees of oxidative stress [11,12].

Oxidative stress is a disruption of the equilibrium between free radicals and antioxidants, with a shift in favor of free radical production. Free radicals are molecules that contain an uneven number of electrons, a property that facilitates reactions between free radicals and other molecules. This triggers large-chain chemical reactions that can damage cell constituents, such as proteins, lipids, and DNA [19]. Oxidative stress compromises the female reproductive system by altering the efficiency of the immune system. Several studies have determined that toxic substances and medications can negatively affect fertility by increasing oxidative stress [20].

When considering the increased incidence of hypertension and infertility, it is important to elucidate the mechanisms and causes of oxidative stress in patients who require antihypertensive medication. Therefore, in our study, we investigated the relationship between oxidative stress and infertility resulting from exposure to antihypertensive medication in Rattus norvegicus. Following the evaluation of serum oxidant MDA levels among the groups, it became apparent that MTL, ALD, and RML increased serum MDA levels significantly when compared to the control group and the other antihypertensive medications investigated in the study. However, serum MDA levels in the CL and RLD groups were similar to the levels seen in the control group. When comparing the serum antioxidant tGSH levels, the CL group levels were similar to those of the control group; however, the serum antioxidant tGSH levels were significantly decreased in the RLD, MTL, ALD, and RML groups.

Various studies have stated that clonidine, in addition to its use as an antihypertensive, can alleviate the symptoms of withdrawal associated with some addictions, including smoking, by acting on the central nervous system [21]. According to a study performed by El-Naga et al., it was reported that clonidine induced a depression-like effect that caused an increase in oxidative stress (decreasing GSH and antioxidant enzyme superoxide dismutase, increasing MDA levels) in Rattus norvegicus brains [22]. Conversely, according to a study by Yusoff et al., who used experimental hypertension-induced Rattus norvegicus models, clonidine decreased oxidative stress and increased antioxidant levels [23]. The differences in the results of these studies are possibly due to the different experimental hypertensive models used.

A study carried out by Elkomy et al. showed that clonidine improves kidney function and decreases inflammation and fibrosis in the kidneys of Rattus norvegicus with induced chronic alcoholism. Clonidine decreases renal oxidative stress by decreasing myeloperoxidase (the enzyme that produces hydrogen peroxide in phagolysosomes), malondialdehyde, inducible nitric oxide synthase, and total nitric oxide levels, while increasing superoxide dismutase levels [24]. According to the data gathered in this study, the increase in MDA levels and decrease in GSH levels observed in the clonidine group were similar to those in the control group, and there were no statistically significant differences; thus, no effect on rat fertility was noted. In a study by Salman et al. investigating the effect of antihypertensive drugs on the oxidant/antioxidant parameters of ovarian tissue, no prominent negative effect was observed after treatment with clonidine and rilmenidine [13]. These findings support our findings, while concluding that the effect of clonidine on oxidative stress remains debatable.

Rilmenidine is an antihypertensive drug that stimulates the effects of the sympathetic system on the central nervous system. According to studies, rilmenidine increased the serum levels of MDA and myeloperoxidase (MPO) in Rattus norvegicus kidney tissue samples more than methyldopa and ramipril, and less than clonidine and amlodipine. Therefore, rilmenidine exerts a nephrotoxic effect due to increased oxidative stress [25,26]. In a study performed by Mercer et al., it was shown that 3,4-metilendioksimetamfetaminin (MDMA) affects serotonin (5-HT) neurons primarily in the primitive brain. It causes degeneration in 5-HT axons and nerve fibers due to mitochondria-mediated oxidative stress; however, rilmenidine completely and selectively protects 5-HT neurons against MDMA-mediated oxidative stress [27]. In a study investigating the effect of antihypertensive drugs, including rilmenidine, on the uterus, rilmenidine was shown to induce moderate negative effects [12]. In this study, serum MDA levels in the clonidine group were similar to those in the control group, and serum tGSH levels were significantly decreased. However, no infertile Rattus norvegicus were present in this group.

Methyldopa is a first-line antihypertensive drug and one of the most commonly used antihypertensive drugs in the world [28]. Mahmud H. et al. investigated the side effects caused by methyldopa by reducing erythrocyte production or causing hemolysis to mimic anemia. Methyldopa causes oxidative stress by reducing the GSH/GSSG ratio, thus causing anemia [29]. In a study by Salman et al., methyldopa increased the levels of MDA and decreased the levels of GSH in uterine and ovarian tissues. In our study, an increase in serum MDA levels and a decrease in serum tGSH levels were found to be statistically significant, causing Rattus norvegicus to become infertile [13]. 

Amlodipine exerts both antihypertensive and antioxidant activities in vivo, and effectively inhibits oxidative stress-mediated cardiovascular damage due to the effects of angiotensin II. According to a study performed by Zhou, M. S. et al., amlodipine decreases blood pressure and aortic hypertrophy, has significant antioxidant effects, and preserves endothelium function in angiotensin-II-induced hypertension [30]. In a study by Ganafa et al., performed using Rattus norvegicus with hypertension-induced by oxidative stress due to glutathione inhibition, it was shown that the antihypertensive effects of amlodipine decreased due to oxidative stress mediated by partial prostanoid endothelium-based factors and nitric oxide [31]. In another study, amlodipine inhibited excessive MDA production and subsequently reduced oxidative stress in atherosclerosis-induced Rattus norvegicus. Amlodipine accelerates erythrocyte glutathione redox cycle activity, thereby increasing the efficacy of the glutathione system [32]. In another study, it was shown that lipophilic calcium channel antagonists inhibited lipid peroxidation by modifying the physicochemical features of the cell membrane lipid bilayers, independent of calcium channel inhibition. It was observed that amlodipine was the most powerful antioxidant among the calcium channel blockers due to its varied biophysical interactions with the lipid bilayer of the cell membrane [33]. In our study, amlodipine caused a statistically significant increase in MDA levels and a decrease in GSH levels when compared with the control group, and caused infertility in two of the six Rattus norvegicus in the group. In a study by Salman et al., who investigated the biochemical side effects of antihypertensive drugs on ovarian tissue, amlodipine was classified as having moderate negative effects. In this study, oxidative stress increased after exposure to amlodipine, in contrast to the significant antioxidant effects reported in the literature [13,34,35,36,37,38]. In our study, amlodipine decreased fertility more than methyldopa and less than ramipril. This finding confirmed our results and showed that oxidative stress may be an indirect parameter.

Ramipril is a powerful antihypertensive drug. It has been shown that ramipril increases endothelium-dependent vasodilation in type 2 diabetic Rattus norvegicus, potentially by decreasing serum ROS levels [39]. Furthermore, it has been shown that ramipril decreases blood pressure and oxidative stress in post-transplant hypertensive patients [40]. In a study investigating the neuroprotective efficacy of ramipril in decreasing white matter lesions due to chronic hypoperfusion and inhibiting oxidative stress, the ramipril group demonstrated significant neuroprotection. Malondialdehyde (MDA) and oxidized glutathione (GSSG)/total glutathione (GSH t) ratios were significantly decreased in the ramipril group. These results show that ramipril can protect against white matter lesions caused by chronic ischemia due to its antioxidant features [41,42]. In our study, the ramipril group had the highest serum MDA levels and lowest serum GSH levels, and ramipril had the greatest oxidative effects. This is in contrast to its purported antioxidant effects reported in the literature [39,40], and in our opinion, this oxidative stress negatively affects fertility to a higher degree than other antihypertensive medications. The ramipril group contained three out of six infertile Rattus norvegicus, the highest ratio of infertility among all the groups. Previous studies [11,12] investigating the effects of chronic antihypertensive medication usage on the oxidant and antioxidant parameters of ovarian and uterine tissues show that ramipril has severe negative effects on both groups. These findings are consistent with our findings.

## 5. Conclusions and Recommendations

We evaluated our results by comparing groups to obtain a robust data set. According to our study, in Rattus norvegicus treated with clonidine, the oxidant and antioxidant parameters and the reproduction test results were similar to those in the control group. Statistically, no significant difference in MDA levels was observed between the CL and RLD groups; however, a significant difference was observed between the MTL, ALD, and RML groups. The highest level of MDA was recorded in the control group, followed by the CL group. We discovered that methyldopa, amlodipine, and ramipril have a negative effect on fertility due to increased oxidative stress and decreased antioxidant levels, thus causing varying degrees of infertility. One Rattus norvegicus from the MTL group did not become pregnant and was deemed infertile. Four Rattus norvegicus became pregnant, and two Rattus norvegicus were assumed to be infertile in the ALD group. The RML group contained the highest number of infertile Rattus norvegicus individuals, with a total of three. In light of these data, the following recommendations are considered appropriate. Oxidative stress is increased in women with infections; thus, antioxidants can be used to successfully treat infertility caused by oxidative stress. L-carnitine (LC) and acetyl L-carnitine (ALC) have huge potential to regulate the oxidative and metabolic state of the female reproductive system. The vulnerability of the female reproductive system to free radicals necessitates new advanced treatments, and for this purpose, the “half vitamins” LC and ALC may be used separately, together, or with other antioxidants. The mean ages for marriage and conception are increasing in our country and worldwide. This situation increases the probability of hypertension and the associated usage of antihypertensive medication as a compounding factor in women with a desire to have children; we believe that public medicine and infertility experts should be aware of this fact. Little information is available in the literature regarding the association between antihypertensive medication and female infertility treatment. For this reason, larger randomized trials are required. A novel field of research is emerging, in which a new group of antihypertensive drugs is being developed that can decrease oxidative stress and nitric acid, thus preventing hypertension and minimizing the complications associated with hypertension.

## Figures and Tables

**Table 1:** Distribution of reproductive test results in all groups.

Reproductive Test Results	Control	CL	RLD	MTL	ALD	RML
	$\bar{X}$ ± SS	$\bar{X}$ ± SS	$\bar{X}$ ± SS	$\bar{X}$ ± SS	$\bar{X}$ ± SS	$\bar{X}$
**Number of pregnant rats**	6.00 ± 1.02	6.00 ± 1.12	6.00 ± 1.05	5.01 ± 0.8	4.00 ± 0.9	3.00 ± 0.8
**Pregnancy duration (days)**	29.83 ± 4.21	33.83 ± 5.21	32.16 ± 5.01	34.8 ± 5.33	38.5 ± 5.92	44.66 ± 6.01
**Infertile rats**	0	0	0	1.00 ± 0.01	2.00 ± 0.22	3.00 ± 1.02
**Number of pups born**	6.83 ± 1.5	6.33 ± 1.4	6.16 ± 1.1	6.20 ± 1.5	5.00 ± 1.0	4.66 ± 0.9
**Pup** **sex** Male Female	24 17	20 18	21 16	18 13	8 12	5 9

**Table 2:** Comparison of serum MDA and tGSH levels between fertile and infertile Rattus norvegicus.

Parameter	Fertility		Test and *p*-Value
	Infertile (n = 6)	Fertile (n = 30)	
MDA (µmol/gr)	4.78 ± 1.30	2.76 ± 1.60	**t = 2.85, *p* = 0.007**
tGSH (nmol/gr)	2.86 ± 1.45	6.75 ± 2.75	**t = 3.35, *p* = 0.002**

The serum MDA and tGSH levels in Rattus norvegicus were compared according to their fertility status, as shown in Table 2. In terms of both MDA and tSGH levels, there was a significant difference between the fertile and infertile groups (*p* = 0.007 and *p* = 0.002, respectively).

## Data Availability

The raw data supporting the conclusions of this article will be made available by the authors on request.
